# Autophagy-Mitophagy Pathway-Linked Genetic Variants Associate with Systemic Inflammation and Interact with Dietary Factors in Asian and European Cohorts

**DOI:** 10.3390/ijms27073062

**Published:** 2026-03-27

**Authors:** Youngjin Choi, Sunmin Park

**Affiliations:** 1 Department of Food Science & Technology, Hoseo University, Asan 31499, Republic of Korea; ojchoi@hoseo.edu; 2 Department of Bioconvergence, Hoseo University, Asan 31499, Republic of Korea; 3 Department of Food and Nutrition, Obesity/Diabetes Research Center, Hoseo University, Asan 31499, Republic of Korea

**Keywords:** systemic inflammation, autophagy, mitophagy, genetic variants, gene–lifestyle interaction, immunity, UK Biobank, KoGES

## Abstract

Autophagy-mitophagy pathways are essential for regulating immune homeostasis. However, their contribution to population-level chronic low-grade systemic inflammation (SI) remains unclear. The objective was to investigate the association between variation in the genes related to the autophagy-mitophagy pathways and SI, and to examine whether lifestyle factors modify this relationship. We conducted genome-wide association studies and gene-set enrichment analyses using data from the Korean Genome and Epidemiology Study (KoGES, n = 28,102) and UK Biobank (UKBB, n = 343,892). SI was defined as an elevated white blood cell count or high-sensitivity C-reactive protein. Using Core Longevity State Vectors (CLSVs)—gene sets representing immune-longevity pathways derived from comparative transcriptomic analysis—we tested six pathways and constructed a weighted genetic risk score (GRS) from significant variants. Gene–lifestyle interactions were examined with respect to major dietary and lifestyle factors. Among six CLSVs, only CLSV-2 (mitophagy and autophagy) showed a significant association with SI (β = 0.425, *p* = 0.008). Six single nucleotide polymorphisms (SNPs) in autophagy-mitophagy genes (*INPP5D*, *ATG16L1*, *ATG7*, *AP3S1*, *OPTN*, and *VPS33A*) were associated with SI in KoGES (*p* < 5 × 10^−5^), and ten SNPs (genes selected in KoGES plus *RAB7A*, *ATG12*, *VPS33A*, *BECN1*) reached genome-wide significance in UKBB (*p* < 5 × 10^−8^). A higher GRS was associated with increased SI in both cohorts and was strongly associated with metabolic syndrome (MetS, OR = 1.91 in KoGES; OR = 1.62 in UKBB). SI was characterized by neutrophilia with relative lymphopenia. In UKBB, significant gene–lifestyle interactions were observed for diet, physical activity, smoking, and alcohol (*p* < 0.01). Favorable lifestyle factors reduced SI most effectively in individuals with protective genotypes. Among individuals with a high vegetable/fruit intake, SI prevalence was 35%, 36%, and 38% in the negative-, zero-, and positive-GRS groups, respectively, compared with 36%, 45%, and 48% in the low-intake groups. In conclusion, genetic variations in autophagy-mitophagy pathways specifically influence SI. Genetic predisposition substantially modifies the benefits of lifestyle, underscoring the importance of integrating genetic and lifestyle factors in understanding SI susceptibility.

## 1. Introduction

Chronic low-grade systemic inflammation (SI), characterized by persistent subclinical elevation of circulating inflammatory markers, is a central biological process underlying aging and the development of chronic diseases, including metabolic syndrome (MetS), cardiovascular disease (CVD), and immune-related disorders [1]. Elevated white blood cell (WBC) counts and C-reactive protein (CRP) levels, even within clinically normal ranges, are widely used markers of SI and have been consistently associated with increased morbidity and mortality [2,3]. Unlike acute inflammatory responses to infection or injury, chronic SI represents ongoing immune activation in the absence of overt pathology, often associated with metabolic dysregulation, cellular stress, and age-related functional decline [4]. Identifying genetic determinants of SI susceptibility is essential for understanding individual variation in inflammation-related disease risk.

Autophagy and mitophagy are evolutionarily conserved cellular quality-control pathways that maintain immune homeostasis by removing damaged organelles and regulating inflammatory signaling [5,6]. Experimental studies demonstrate that impaired autophagy or mitophagy enhances inflammatory responses and accelerates aging, whereas activation of these pathways suppresses pro-inflammatory cytokine production and extends healthspan [7]. In humans, autophagy capacity declines with age and is further compromised by obesity and metabolic stress, contributing to chronic SI [7]. Genetic variation in autophagy- and mitophagy-related genes may therefore influence individual susceptibility to chronic inflammatory states by modulating cellular quality-control efficiency. However, population-based genetic evidence linking these pathways to SI remains limited.

Genome-wide association studies (GWAS) have identified numerous loci associated with inflammatory markers [8,9]. However, individual variants typically account for only a small proportion of phenotypic variance. Weighted genetic risk scores (GRS), which aggregate effects across multiple variants, provide a more powerful approach to capture cumulative genetic contributions to complex traits [10]. Pathway-based gene-set analyses further enhance biological interpretability by prioritizing functionally related genes over isolated loci [11]. Despite accumulating evidence linking autophagy and mitophagy to immune regulation, the contribution of genetic variation in these pathways to population-level SI remains poorly characterized.

Lifestyle factors—including physical activity, smoking, and dietary patterns—are established modulators of both autophagy-related processes and inflammatory status [12]. Physical activity activates autophagy and mitophagy across multiple tissues [13], while smoking and unhealthy diets exacerbate oxidative stress and inflammatory signaling [14]. Genetic susceptibility related to autophagy and mitophagy may therefore interact with lifestyle behaviors to influence inflammatory phenotypes; however, empirical evidence examining such gene–lifestyle interactions remains scarce. Most previous studies focus on genetic effects on metabolic disease risk in single populations. Evidence for interactions between genetic risk and dietary or lifestyle factors remains limited and inconsistent, and direct comparisons between Asian and European cohorts are scarce, representing a gap in understanding population-specific gene–environment interactions.

The present study examined associations between an autophagy- and mitophagy-related GRS derived from pathway-based gene-set analysis and SI, defined by elevated WBC count and CRP levels. To enhance robustness and generalizability, findings were evaluated in two large, independent population-based cohorts: the Korean Genome and Epidemiology Study (KoGES) and the UK Biobank (UKBB). In addition, potential modification of genetic associations with SI by lifestyle factors was assessed, providing insights into the interplay between genetic susceptibility, lifestyle behaviors, and inflammatory phenotypes.

## 2. Results

### 2.1. Baseline Characteristics According to SI Status

Participants with high-SI were slightly younger than those with low SI in both cohorts (KoGES: 54.1 vs. 54.6 years; UKBB: 55.7 vs. 56.3 years; *p* < 0.001; Table 1). Gender distribution differed significantly, with more men in the high-SI group in the KoGES (43.5% vs. 30.5) and also in the UKBB (48.8% vs. 46.6%; *p* < 0.001). Anthropometric measures were consistently elevated in the high-SI group. BMI was significantly higher (KoGES: 24.3 vs. 23.8 kg/m^2^; UKBB: 27.0 vs. 26.3 kg/m^2^; *p* < 0.001), as was body fat percentage. The prevalence of MetS was markedly higher in the high-SI group than in the low-SI group (KoGES: 20.8% vs. 11.7%; UKBB: 27.4% vs. 16.8%; *p* < 0.001). As expected, inflammatory biomarkers were substantially elevated in the high-SI group, with significantly higher WBC counts (KoGES: 7.36 ± 0.013 vs. 5.13 ± 0.009 × 10^9^/L; UKBB: 8.34 ± 0.01 vs. 5.77 ± 0.01 × 10^9^/L) and serum hsCRP concentrations (KoGES: 0.24 ± 0.006 vs. 0.091 ± 0.004 mg/L; UKBB: 1.28 ± 0.003 vs. 1.07 ± 0.002 mg/L) (all *p* < 0.001). Lifestyle factors were significantly associated with SI status. The high-SI group reported lower alcohol consumption (<7.9 g/day), lower physical activity (<30 min moderate activity/day), and substantially higher smoking prevalence (KoGES: 20.0% vs. 6.9%; UKBB: 15.6% vs. 5.9%; *p* < 0.001).

### 2.2. Association of SI with Metabolic Outcomes and WBC Subtypes

**Figure 1:**
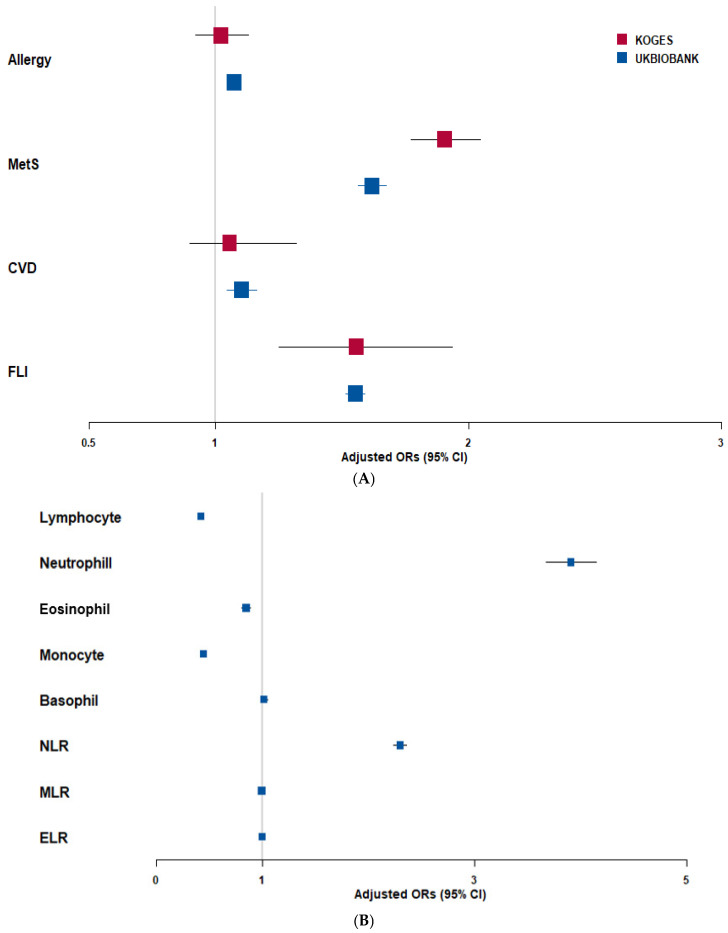
Adjusted odds ratios (ORs) and 95% confidence intervals (CI) of chronic low-grade systemic inflammation (SI) by metabolic disorders. (**A**) Association of SI with metabolic syndrome (MetS), allergy, cardiovascular diseases (CVD), and fatty liver index (FLI) in the Korean Genome and Epidemiology Study (KoGES) and the UK Biobank (UKBB) cohorts. (**B**) Association of SI with subtypes of white blood cells and the neutrophils to lymphocytes ratio (NLR), monocytes to lymphocytes ratio (MLR), and eosinophils to lymphocytes ratio (ELR) in the UKBB. Cutoffs: 60 for FLI; 70% for neutrophils; 20% for lymphocytes; 6% for eosinophils; 10% for monocytes; 1% for basophils; 3 for NLR; 0.35 for MLR; and 0.05 for ELR.

High-SI was consistently associated with adverse metabolic outcomes (Figure 1A). The odds ratios (ORs) of MetS (KoGES: OR = 1.91, 95% confidence intervals (CI): 1.77–2.05; UKBB: OR = 1.62, 95% CI: 1.56–1.68) and FLI (OR = 1.56 and 1.55, respectively; all *p* < 0.001) were significantly elevated in the high-SI group. Associations with CVD and allergy were weak or inconsistent, showing marginal significance in UKBB but not in KoGES. In UKBB, SI was characterized by alterations in distinct WBC subtypes (Figure 1B). High-SI showed strong positive associations with neutrophil counts (OR = 3.91, 95% CI: 3.68–4.15) and neutrophil-to-lymphocyte ratio (NLR; OR = 2.31), whereas lymphocyte, monocyte, and eosinophil counts were inversely associated (OR = 0.42, 0.85, and 0.45, respectively; all *p* < 0.001). Basophil counts showed no significant association. Therefore, SI is primarily characterized by neutrophilia with relative lymphopenia, consistent with a pro-inflammatory myeloid-skewed immune phenotype.

### 2.3. Association of Core Longevity State Vector (CLSV) Gene Sets with SI

Among the six CLSVs, only CLSV-2 (mitophagy and autophagy genes) showed a significant association with SI (β = 0.425, *p* = 0.008; Table 2). CLSV-1 (damage tolerance), CLSV-3 (proteostasis), CLSV-4 (basal immune readiness), CLSV-5 (inflammasome restraint), and CLSV-6 (resolution capacity) were not significantly associated with SI (all *p* > 0.05). These results indicate that mitophagy and autophagy pathways, rather than other longevity-related immune programs, are specifically linked to SI.

### 2.4. Genetic Variants in CLSV-2 Genes Associated with SI

Following MAGMA gene-set enrichment analysis identifying CLSV-2 (autophagy-mitophagy) as significantly associated with SI, we identified genetic variants within this pathway in both cohorts (Table 3).

Discovery in KoGES: Six independent SNPs in autophagy-mitophagy genes were identified at the relaxed significance threshold (*p* < 5 × 10^−5^): rs68147208 in Inositol Polyphosphate-5-Phosphatase D (*INPP5D*), rs368068803 in Autophagy Related 16 Like 1 (*ATG16L1*), rs149819403 in Autophagy Related 7 (*ATG7*), rs10055640 in Adaptor Related Protein Complex 3 Subunit Sigma 1 (*AP3S1*), rs76421222 in Optineurin (*OPTN*), and rs76696405 in Vacuolar Protein Sorting-Associated Protein 33A (*VPS33A*) (Table 3A). These variants showed modest effect sizes (OR range: 0.81–1.27) and were primarily located in intronic or regulatory regions, consistent with their potential roles in gene regulation. The six SNPs, located across six different genes, were used to construct the KoGES genetic risk score.

Replication in UKBB: Within the predefined CLSV-2 gene regions, ten SNPs reached genome-wide significance (*p* < 5 × 10^−8^): rs7559281 (*INPP5D*), rs9848833 (*ATG7*), rs35904610 and rs9821206 (Ras-Related Protein Rab-7a, *RAB7A*), rs7724740 (Autophagy Related 12, *ATG12*), rs10075090 (Sequestosome 1, *SQSTM1*), rs73421728 (*VPS33A*), rs2925352 (Vacuolar Protein Sorting-Associated Protein 18 Homolog, *VPS18*), rs9972681 (Microtubule-Associated Protein 1 Light Chain 3 Beta, *MAP1LC3B*), and rs9891429 (Beclin 1, *BECN1*) (Table 3B). These variants demonstrated stronger statistical support than the KoGES discovery SNPs and showed generally protective associations with SI (OR range: 0.51–1.08), reflecting the larger sample size (n = 343,892 vs. n = 30,599), which enabled the detection of variants with smaller effect sizes. The ten genome-wide significant SNPs were used to construct the UKBB genetic risk score.

Cross-cohort gene-level replication. Three genes identified in KoGES—*INPP5D*, *ATG7*, and *VPS33A*—harbored genome-wide significant SNPs in UKBB, confirming gene-level replication across ethnically distinct populations (East Asian and European). However, the specific lead SNPs differed between cohorts (different rsIDs and genomic positions within the same genes; Table 3), reflecting population-specific linkage disequilibrium structure and allele frequency differences. This gene-level replication pattern, where the same genes but different SNPs show associations across populations, is consistent with shared biological mechanisms underlying SI despite population-specific genetic architecture. The identification of additional autophagy-mitophagy genes in UKBB (*RAB7A*, *ATG12*, *SQSTM1*, *VPS18*, *MAP1LC3B*, *BECN1*) further supports the pathway-level association and highlights the comprehensive involvement of autophagy-mitophagy processes in SI regulation.

### 2.5. Associations of the GRS with Disease Outcomes

**Figure 2:**
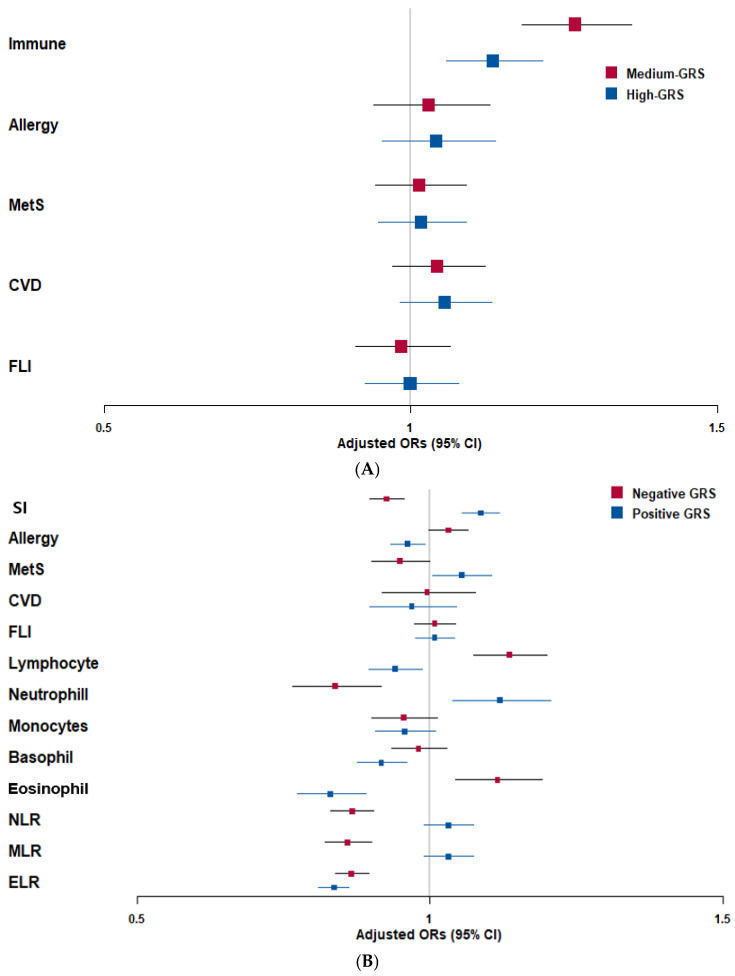
Adjusted odds ratios (ORs) and 95% confidence intervals (CI) of genetic risk scores (GRS) with chronic low-grade systemic inflammation (SI) risk in the Korean Genome and Epidemiology Study (KoGES) and the UK Biobank (UKBB). (**A**) Association of GRS with metabolic syndrome (MetS), allergy, cardiovascular diseases (CVD), and fatty liver index (FLI) in the Korean Genome and Epidemiology Study (KoGES). (**B**) Association of GRS with MetS, allergy, CVD, FLI, subtypes of white blood cells, and the neutrophils to lymphocytes ratio (NLR), monocytes to lymphocytes ratio (MLR), and eosinophils to lymphocytes ratio (ELR) in the UKBB Cutoffs: 60% for FLI; 70% for neutrophils; 20% for lymphocytes; 6% for eosinophils; 10% for monocytes; 1% for basophils; 3 for NLR; 0.35 for MLR; 0.05 for ELR.

In the KoGES, participants were stratified into tertiles of the weighted GRS derived from the CLSV-2 SNPs. Compared with the lowest tertile (reference), the medium and high GRS tertiles showed progressively higher risks of SI (Figure 2A). Similar, though weaker, trends were observed for allergy, MetS, CVD, and FLI. However, these did not consistently reach statistical significance. In the UKBB, participants were categorized into negative GRS (protective), zero GRS (reference), and positive-GRS (risk) groups based on the skewed GRS distribution. The positive-GRS group exhibited significantly elevated odds of SI, MetS, and CVD than the zero-GRS group (Figure 2B). In contrast, the negative-GRS group showed generally lower risks, indicating protective genetic effects. In the UKBB, analysis of immune cell traits revealed that a positive GRS was associated with higher neutrophil counts and NLR, while a negative GRS was associated with elevated lymphocyte and eosinophil levels. These findings suggest that genetic variation in mitophagy/autophagy pathways influences both SI and immune cell composition.

### 2.6. Gene–Lifestyle Interactions in SI

**Figure 3:**
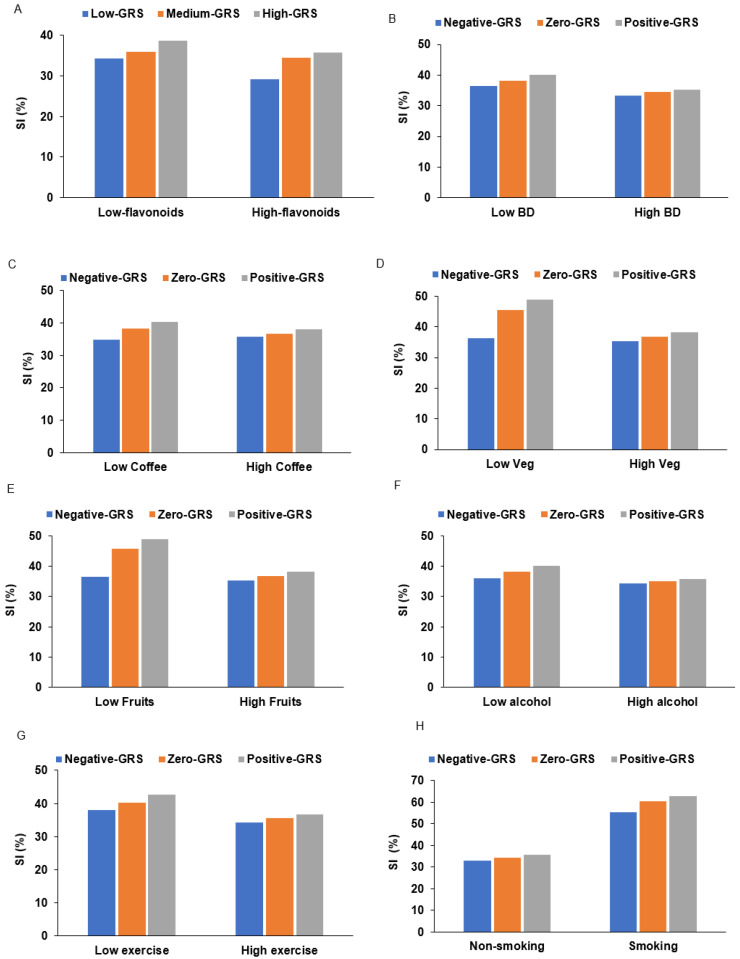
The percentage of participants with chronic low-grade systemic inflammation (SI) according to genetic risk score (GRS) groups for SI in the Korean Genome and Epidemiology Study (KoGES) and the UK Biobank (UKBB). (**A**) The percentage of SI according to the low- and high-flavonoid groups in the KoGES (cutoff: 42 mg/day). (**B**) The percentage of SI in the balanced diet pattern in the UKBB (cutoff: 66th percentile). (**C**) The percentage of SI in the low- and high-coffee in the UKBB (cutoff: 1 cup/day). (**D**) The percentage of SI in the low- and high-vegetable groups in the UKBB (cutoff: 1 serving/day). (**E**) The percentage of SI in the low- and high-fruit groups in the UKBB (cutoff: 1 serving/day). (**F**) The percentage of SI in the low- and high-alcohol groups in the UKBB (cutoff: 7.9 g/day). (**G**) The percentage of SI in the low- and high-exercise groups in the UKBB (cutoff: 30 min/day). (**H**) The percentage of SI in the non-plus former-smoking and smoking groups in the UKBB. Covariates included age, sex, education, residential area, body mass index (BMI), physical activity, smoking, alcohol consumption, and medication use. Any variable identical to an independent variable was excluded from the covariates. Significantly different among the GRS groups in low- and high categories in each figure at *p* < 0.001 by chi-square test.

Categorical GRS x lifestyle interactions: In the KoGES, interactions between GRS and lifestyle factors were examined (Table 4A). Among dietary and lifestyle variables, only flavonoid intake showed nominal interaction with GRS (*p* = 0.037), while fat intake showed borderline significance (*p* = 0.053); neither passed the Bonferroni correction. The prevalence of SI was lower among participants in the low-GRS group with high flavonoid intake (>42 mg/day) than those with low flavonoid intake (Figure 3A). However, this protective effect was attenuated in the medium- and high-GRS groups, suggesting that flavonoid benefits are primarily evident among those with low genetic risk. In contrast, the UKBB demonstrated multiple significant gene–lifestyle interactions (Table 4B). Significant interactions were detected for dietary balance (*p* = 0.0053), coffee intake (*p* = 0.0004), total vegetable and fruit intake (*p* < 0.0001), vegetable intake (*p* < 0.0001), fruit intake (*p* = 0.0003), alcohol consumption (*p* = 0.0034), physical activity (*p* = 0.0063), and smoking status (*p* = 0.0013). In contrast, meat intake showed no interaction (*p* = 0.204).

SI prevalence increased stepwise from negative- to positive-GRS groups across all lifestyle factors (Figure 3B–D). For vegetable and fruit intake, low consumption was associated with SI prevalence of 36%, 45%, and 48% in the negative-, zero-, and positive-GRS groups, respectively, while high intake reduced SI to 35%, 36%, and 38%, with the greatest benefit in the negative-GRS group. A low intake of alcohol was associated with SI prevalence of 35%, 38%, and 40% across GRS groups, whereas a high intake was associated with modest increases of 33%, 35%, and 36%. Higher dietary balance, coffee intake, and physical activity were consistently associated with lower SI prevalence, with the strongest effects in the negative-GRS group. Overall, favorable lifestyle behaviors were associated with lower SI across all genetic risk categories. However, the protective effects progressively diminished from the negative- to the positive-GRS groups. Notably, individuals with positive GRS maintained elevated SI prevalence even under optimal lifestyle conditions, indicating that genetic predisposition in autophagy-mitophagy pathways substantially modifies the impact of lifestyle factors on SI.

Continuous GRS × lifestyle interactions: To maximize statistical power and avoid arbitrary categorization thresholds, we conducted interaction analyses using continuous GRS and lifestyle measures (Table S1). In UKBB, this approach revealed four significant dietary interactions: coffee consumption (β = −0.010, 95% CI: −0.018 to −0.003, *p* = 0.006), total fruit intake (β = −0.004, 95% CI: −0.008 to −0.0002, *p* = 0.039), combined vegetable and fruit intake (β = −0.002, 95% CI: −0.004 to −0.0001, *p* = 0.040), and meat consumption (β = +0.023, 95% CI: 0.013–0.034, *p* < 0.001). Notably, meat showed a highly significant interaction in continuous analysis (*p* < 0.001) but not in categorical analysis (*p* = 0.204), demonstrating that continuous measures can detect dose–response relationships that may be obscured by categorization. Coffee showed the strongest protective interaction, with each additional cup per day reducing genetic susceptibility by 0.010 log-odds units. In contrast, meat consumption showed a strong adverse interaction, with higher intake amplifying genetic risk. Total vegetable intake alone did not show a significant interaction (β = −0.002, *p* = 0.224), though combined vegetable and fruit intake did (*p* = 0.040), suggesting synergistic effects or fruit-driven interactions. Physical activity and alcohol consumption showed independent protective main effects (both *p* < 0.001) but did not significantly modify genetic risk in continuous analyses (*p* = 0.126 and *p* = 0.305, respectively). It indicates that these factors influence SI through mechanisms independent of autophagy-mitophagy genetic pathways.

### 2.7. Sensitivity Analyses Confirm Robustness of Genetic Findings

To address potential concerns about threshold-dependent effects and cross-cohort comparability, we conducted comprehensive sensitivity analyses in KoGES (Supplementary Table S2A–C). Using the continuous GRS, associations with SI remained significant across multiple binary definitions: WBC-only SI (OR = 1.19, 95% CI: 1.12–1.27, *p* < 0.001) and quartile-based SI derived independently of metabolic syndrome (OR = 1.10, 95% CI: 1.03–1.17, *p* < 0.01). CRP-only SI (prevalence 1.72%) showed no significant association due to limited statistical power in this East Asian cohort, where baseline CRP levels are substantially lower (median 0.063 mg/L, IQR: 0.040–0.118) (Table S2A).

To eliminate threshold artifacts from both genetic risk categorization and inflammatory marker definition, we analyzed continuous GRS associations with continuous inflammatory markers using linear regression. Each unit increase in continuous GRS was associated with significantly elevated WBC (2.23 × 10^9^/L, 95% CI: 2.20–2.26, *p* < 0.001) and CRP (1.54-fold higher, 95% CI: 1.49–1.59, *p* < 0.001) in KoGES. These continuous associations replicated in UKBB (WBC: 2.57 × 10^9^/L, 95% CI: 2.56–2.58; CRP: 1.20-fold higher, 95% CI: 1.19–1.21; both *p* < 0.001), demonstrating that autophagy-mitophagy genetic variants robustly influence systemic inflammation across ethnically distinct populations independent of how inflammation is operationalized or how genetic risk is categorized (Table S2B).

To address whether defining SI thresholds based on MetS-associated values—then reporting SI-MetS associations—introduced circularity, we tested this relationship using quartile-based SI (top 25% of WBC or CRP distribution) derived independently of any metabolic analyses. This yielded nearly identical results to the primary analysis (OR = 1.63, 95% CI: 1.52–1.74 vs. OR = 1.65, 95% CI: 1.52–1.78; MetS prevalence 21.4% vs. 20.8%), demonstrating that the relationship is biological rather than methodological (Table S2C). Collectively, these analyses confirm that genetic associations with SI are robust regardless of threshold choice or GRS operationalization (categorical vs. continuous).

## 3. Discussion

SI is a central biological process linking aging, metabolic disorders, and CVD [15]. While previous GWAS have identified loci associated with individual inflammatory biomarkers such as circulating CRP and WBC count [2,3], these studies have primarily focused on downstream immune signaling pathways, including cytokine regulators such as interleukin (IL)6, IL1B, and Signal Transducer and Activator of Transcription 3 (STAT3) [9,16]. Polygenic risk scores derived from these loci have been associated with cardiometabolic diseases and mortality [16], thereby establishing the heritability of traits associated with inflammation. However, a critical gap remains: the contribution of upstream cellular quality-control pathways—particularly autophagy and mitophagy—to population-level systemic inflammation has not been systematically characterized.

Experimental evidence strongly supports a mechanistic role for autophagy and mitophagy in regulating SI [17]. Defective autophagy and mitophagy promote chronic inflammation by enabling the accumulation of damaged mitochondria and damage-associated molecular patterns (DAMPs), which activate inflammasomes and nuclear factor κB (NF-κB) signaling pathways [18,19]. Genetic ablation of autophagy genes (*ATG5*, *ATG7*, *BECN1*) in animal models has been shown to result in spontaneous inflammation and accelerated aging [20]. The pharmacological activation of autophagy suppresses inflammatory responses [21]. Caloric restriction, exercise, and dietary polyphenols have been shown to exert anti-inflammatory effects through autophagy activation [19,20]. Despite this compelling mechanistic foundation, population-based genetic studies linking autophagy and mitophagy pathways to SI and their interactions with lifestyle factors remain limited. One key reason is that autophagy-related genes typically harbor multiple variants with small individual effects that fail to reach genome-wide significance in conventional GWAS, making pathway-level aggregation essential for detection [22,23].

The present study addresses these gaps by integrating pathway-based gene-set analysis with large-scale genetic and lifestyle data in two independent cohorts. Using CLSVs derived from comparative bat-human transcriptomics [17,24], six immune-longevity pathways were tested. Only CLSV-2 (mitophagy-autophagy) showed a significant association with SI (β = 0.425, *p* = 0.008). This contrasts with previous GWAS that identified cytokine-centric loci but failed to detect autophagy pathways [9,22], probably because individual autophagy gene variants require pathway-level aggregation for detection [25]. A weighted GRS constructed from CLSV-2 genes demonstrated consistent associations with SI and MetS across Asian (KoGES) and European (UKBB) ancestries. Key genes (*INPP5D*, *ATG7*, *VPS33A*) replicated across cohorts, extending previous inflammatory GWAS by implicating upstream mitochondrial quality-control mechanisms rather than downstream cytokine regulation [9,23,24]. This was consistent with the growing recognition that cellular homeostasis pathways are central to inflammaging [26,27].

A major finding of this study is that dietary factors, but not other lifestyle behaviors, significantly modify genetic susceptibility to SI conferred by autophagy-mitophagy pathway variants. In UKBB, significant interactions were detected for coffee consumption (β = −0.010, *p* = 0.006), fruit intake (β = −0.004, *p* = 0.039), combined vegetable-fruit intake (β = −0.002, *p* = 0.040), and meat consumption (β = 0.023, *p* < 0.001). In contrast, physical activity and alcohol consumption showed strong independent protective effects (both *p* < 0.001) but did not significantly modify genetic risk (*p*-interaction > 0.1), indicating that these factors influence SI through pathways independent of autophagy-mitophagy genetics. This selective interaction pattern is consistent with experimental evidence that dietary components directly modulate autophagy flux: plant-derived polyphenols activate autophagy through AMPK and SIRT1 pathways [28], while dietary protein activates mTOR, the master negative regulator of autophagy [29]. Coffee polyphenols have been shown to induce autophagy and enhance mitochondrial quality control in vitro and in vivo [30], while amino acid abundance from meat consumption potently activates mTORC1 and suppresses ULK1-mediated autophagy initiation [31]. These gene-diet interactions suggest that individuals with genetic impairment in autophagy-mitophagy pathways may benefit from dietary interventions that enhance autophagic flux (increased plant-based foods, coffee) and avoid autophagy suppression (reduced meat intake). Gene–lifestyle interactions were not detected in KoGES, likely reflecting insufficient statistical power in the smaller cohort (n = 30,599 vs. n = 343,892), as interaction detection requires substantially larger sample sizes than main effect analyses [32]. Despite this, categorical analyses in KoGES showed directionally consistent borderline interactions for flavonoid intake (*p* = 0.037) and fat intake (*p* = 0.053), supporting the biological plausibility of the dietary modulation of autophagy-related genetic risk across populations.

This study has several strengths, including large sample sizes in two independent cohorts with different ancestries (Korean and European), pathway-based gene-set enrichment analysis capturing collective effects of functionally related variants, cross-population replication at the gene level, and comprehensive assessment of gene–lifestyle interactions in the adequately powered UKBB cohort. The integration of comparative transcriptomics-derived gene sets (CLSVs) with human GWAS data represents a novel approach linking evolutionary biology to human disease genetics.

Several limitations warrant consideration. First, the observational design precludes causal inference; Mendelian randomization or functional validation studies would strengthen causal claims. Second, SI was defined using clinical biomarkers (circulating WBC and hsCRP levels) rather than direct molecular measures of inflammatory signaling pathways, potentially capturing heterogeneous inflammatory states. Third, dietary and lifestyle variables were self-reported and subject to measurement error and recall bias, which may attenuate the interaction effect. Fourth, although findings were replicated across two large cohorts, population-specific genetic architecture, environmental exposures, and gene-environment correlations may influence effect sizes and limit generalizability to other ancestries. Fifth, the relaxed significance threshold (*p* < 5 × 10^−5^) in the KoGES may have captured false positives, though replication of key genes in the UKBB at genome-wide significance mitigates this concern [33]. Finally, the use of medications such as metformin and rapamycin analogs might directly affect autophagy [34], which was not accounted for, and could confound associations.

Several research priorities emerge from these findings. First, replication in additional independent cohorts with diverse ancestries and harmonized dietary assessment is needed to establish generalizability. Second, functional validation studies should examine autophagy flux and mitophagy activity in human cells or tissues stratified by genotype, linking genetic variation to cellular phenotypes and inflammatory signaling. Third, prospective cohort studies should assess whether autophagy-mitophagy genetic risk predicts incident inflammatory diseases, cardiovascular events, and metabolic disorders beyond cross-sectional biomarker associations. Fourth, intervention trials testing whether dietary or pharmacological autophagy enhancement—such as spermidine supplementation or caloric restriction mimetics—differentially reduces inflammation based on genetic risk profiles, are needed to establish clinical utility. Integration with multi-omics data, including transcriptomics, proteomics, and metabolomics, may further elucidate how genetic variation in autophagy pathways translates into inflammatory phenotypes at the molecular level.

## 4. Materials and Methods

### 4.1. Study Populations

All study procedures were approved by the Institutional Review Boards of the Korean National Research Institute of Health for KoGES (KBP-2015-055) and Hoseo University Ethics Committee (1041231-150811-HR-034-01), the UKBB Ethics Committee (11/NW/0382), and the Hoseo University Ethics Committee (1041231-240820-BR-183-01) in accordance with the Declaration of Helsinki. Written informed consent was obtained from all participants in both cohorts. The KoGES is a large-scale prospective cohort established to identify genetic and environmental risk factors for chronic diseases in the Korean population and to provide evidence for disease prevention and personalized medicine [35]. Participants aged 40–79 years were voluntarily recruited from designated hospitals in major urban centers between 2010 and 2014. The UKBB is a prospective population-based cohort comprising 502,173 participants aged 40–69 years recruited throughout the United Kingdom between 2006 and 2010 [10]. In both cohorts, participants were excluded if they met any of the following criteria: (1) evidence of hypo-immunity, defined as WBC count < 4.0 × 10^9^/L (KoGES: n = 17,927; UKBB: n = 10,227); or (2) missing data on WBC, high-sensitivity CRP (hsCRP); or genetic variants required for GRS construction (KoGES: n = 12,672; UKBB: n = 126,688). In UKBB, analyses were restricted to participants of European ancestry, excluding individuals of other ancestries (n = 21,366; Figure S1B). After applying these exclusion criteria, the final analytical sample comprised 28,102 Korean participants from KoGES (Figure S1A) and 343,892 participants of European ancestry from UKBB (Figure S1B).

### 4.2. Anthropometric and Biochemical Measurements

Demographic and lifestyle information was collected using standardized, validated questionnaires in both cohorts [35,36]. Anthropometric measurements, including body weight, height, and body fat, were obtained with participants wearing lightweight gowns without footwear. Body mass index (BMI) was calculated using the formula BMI = body weight (kg)/(height in m^2^). After a minimum 12 h overnight fast, venous blood samples were collected into heparinized tubes for biochemical analyses. In the KoGES, serum hsCRP concentrations were measured by an enzyme-linked immunosorbent assay. Total WBC count was quantified using automated hematology analyzers. In the UKBB, biochemical assays were performed at a centralized laboratory (UK Biobank Coordinating Centre, Stockport, UK) using standardized protocols with rigorous quality-control procedures [37]. Total WBC count and differential counts, including lymphocytes, neutrophils, monocytes, eosinophils, and basophils, were quantified using automated hematology analyzers (Beckman Coulter LH750, Brea, CA, USA). Serum hsCRP concentrations were measured using immunoturbidimetric high-sensitivity assays (Beckman Coulter AU5800, Brea, CA, USA). Serum triglyceride and γ-glutamyl transferase (γ-GTP) concentrations were measured using enzymatic colorimetric assays on automated clinical chemistry analyzers in both cohorts. The fatty liver index (FLI) was calculated using BMI, waist circumference, serum triglycerides, and γ-GTP concentration [38].

### 4.3. Definition of SI

Participants were classified according to chronic low-grade systemic inflammatory status based on circulating WBC count and hsCRP levels. Individuals with hypo-immunity (WBC < 4.0 × 10^9^/L) were excluded to focus analyses on hyper-immune inflammatory states. In the KoGES, SI cases were defined as participants with a WBC count > 6.2 × 10^9^/L or hsCRP > 1.0 mg/L (n = 10,020; high-SI group), whereas controls had a WBC count between 4.0 and 6.2 × 10^9^/L and hsCRP ≤ 1.0 mg/L (n = 18,082; low-SI group). In the UKBB, SI cases were defined as those with a WBC count > 7.0 × 10^9^/L or hsCRP > 3.0 mg/L (n = 127,185), and controls had a WBC count between 4.0 and 7.0 × 10^9^/L and hsCRP ≤ 3.0 mg/L (n = 216,707). These cutoff values were determined based on thresholds associated with increased risk of MetS within each cohort [2,39,40,41]. Different thresholds were applied between the two cohorts to account for population-specific differences in the distribution of inflammatory markers and their associations with metabolic outcomes [42,43,44].

Sensitivity analyses for SI definition: To assess robustness of genetic findings independent of threshold selection and GRS operationalization, we conducted sensitivity analyses in KoGES testing: (1) alternative binary SI definitions including WBC-only (WBC > 6.2 × 10^9^/L), CRP-only (CRP > 1.0 mg/L), and quartile-based SI (WBC > 6.51 × 10^9^/L or CRP > 0.118 mg/L, representing the 75th percentile of the population distribution); (2) continuous GRS associations with continuous inflammatory markers (WBC and log-transformed CRP) as outcomes using linear regression to eliminate threshold-related artifacts from both the exposure and outcome; and (3) quartile-based SI-metabolic syndrome associations to assess potential circularity, where the quartile definition was derived purely from inflammatory marker distributions without reference to metabolic outcomes. For continuous outcome analyses, linear regression models were fitted with log-transformed inflammatory markers as dependent variables and continuous GRS as the independent variable, adjusted for the same covariates as the primary analysis. β coefficients represent the change in log-transformed marker levels per unit increase in GRS. Cross-cohort validation was performed by examining the associations of continuous GRS with continuous inflammatory markers in both KoGES and UKBB.

### 4.4. Dietary Intake and Lifestyle Assessment

Daily energy intake and nutrient composition were estimated from dietary data obtained through semi-quantitative food frequency questionnaires (SQFFQ) [35,36]. Dietary patterns were derived using principal component analysis with orthogonal rotation (Varimax) to enhance interpretability [2]. Twenty-nine predefined food groups were included as independent variables, and factors were extracted based on eigenvalues > 1.5 [45]. Food groups with absolute factor-loading values ≥ 0.40 were considered to contribute meaningfully to each dietary pattern. Individual pattern scores were calculated by summing the standardized intake of food groups weighted by their corresponding factor loadings. The estimated energy requirement (EER) was calculated using age, sex, body weight, height, and physical activity level, according to ethnicity-specific dietary reference intakes (DRIs) [46,47]. Low and high vegetable, fruit, and coffee intakes were categorized with the cutoffs of 1 serving/day.

Smoking status was categorized as: current smokers (individuals who smoked > 20 cigarettes in the previous 6 months), former smokers (quit ≥ 6 months prior), and never-smokers. Daily alcohol consumption was assessed based on drinking frequency and amount and classified as: low consumer (<7.9 g/day) and high consumers (≥7.9 g/day) [39]. Coffee and tea consumption were evaluated similarly and categorized into tertiles of daily intake. Regular physical activity was defined as moderate-intensity exercise lasting ≥ 30 min per session for ≥3 days per week, and participants meeting this criterion were classified as the high-exercise group [2].

### 4.5. Definition of CLSVs

Gene sets representing immune–longevity pathways were defined based on previous comparative transcriptomic analysis of bat and human immunity [24,48] and operationalized as six Core Longevity State Vectors (CLSVs): CLSV-1 (damage tolerance), CLSV-2 (mitophagy and autophagy), CLSV-3 (proteostasis), CLSV-4 (basal immune readiness), CLSV-5 (inflammasome restraint), and CLSV-6 (resolution capacity). Each CLSV comprised a curated list of functionally related genes and was used as a predefined gene set in pathway-based analyses.

### 4.6. Genotyping and Quality Control

In KoGES, genomic DNA was genotyped using the Korea Biobank Array (Affymetrix “Korean Chip”) provided by the Korea Disease Control and Prevention Agency (KDCA), yielding ~833,000 directly genotyped variants. Genotype imputation was performed using the 1000 Genomes Project Phase 3 East Asian reference panel to increase genomic coverage. Imputation was performed using IMPUTE2. Post-imputation quality control retained variants with an imputation quality score (INFO) ≥ 0.8. Quality control procedures for genotyped and imputed variants followed standard protocols [32]. In UKBB, genome-wide genotyping was performed using the UK BiLEVE Axiom Array or the UKBB Axiom Array [30]. Genotype imputation was performed by UKBB using IMPUTE4 with the Haplotype Reference Consortium (HRC) and UK10K combined reference panels, providing about 96 million imputed variants [41].

Single-nucleotide polymorphisms (SNPs) were retained if they met the following criteria: call rate ≥ 98%, heterozygosity < 30%, missing rate < 4%, Hardy–Weinberg equilibrium (HWE) *p* > 0.05, and no evidence of sex-related genotype bias. SNPs with minor allele frequency (MAF) < 0.005 or HWE *p* < 0.05 were excluded. For imputed variants, an additional INFO score threshold of ≥0.8 was applied to ensure imputation quality. To minimize linkage disequilibrium (LD), SNPs in strong LD were pruned using an r^2^ threshold < 0.2, retaining relatively independent variants for analysis.

To minimize confounding by population stratification, analyses were restricted to genetically homogeneous populations. In KoGES, all participants were of Korean ancestry, representing a genetically homogeneous East Asian population. In UKBB, analysis was restricted to individuals of White British ancestry using self-reported ethnicity and genetic principal component-based classification (field 22006), which employs the first four genetic principal components to identify and exclude ancestry outliers, ensuring a genetically homogeneous European sample.

Population stratification in the discovery phase was quantitatively assessed using genomic inflation calculated from the KoGES genome-wide association study. The genomic inflation factor was λ = 1.032, well below the conventional threshold of 1.05, indicating negligible confounding by population stratification (Figure S2). The quantile-quantile (QQ) plot demonstrates close alignment with the expected null distribution, with deviation only in the tail corresponding to true genetic associations. This low inflation confirms that ancestry restriction combined with demographic and geographic covariates adequately controlled for population structure without requiring genome-wide principal components as additional GWAS covariates.

### 4.7. GWAS and Gene-Set Analysis

A GWAS of SI was conducted in the KoGES discovery cohort using PLINK v2.0 [10] under an additive genetic model. The analysis adjusted for age, sex, education level, residential area, BMI, medication use (antihypertensive, antidiabetic, and lipid-lowering agents), energy and alcohol intake, smoking status, and physical activity level. GWAS summary statistics were analyzed using Multi-marker Analysis of GenoMic Annotation (MAGMA v1.08) [49] to perform gene-based association tests and gene-set enrichment analyses. Each CLSV gene set (CLSV-1 through CLSV-6) was tested for enrichment of associations with SI. Based on the MAGMA results, CLSV-2 (mitophagy and autophagy) showed significant enrichment and was selected for the construction of the GRS. Genes within CLSV-2 were further classified into autophagy-related and mitophagy-related functional groups.

### 4.8. SNP Selection and Weighted GRS Construction

Discovery phase (KoGES): Following the identification of CLSV-2 (autophagy-mitophagy) as significantly associated with SI, SNPs were mapped to the 19 genes comprising this pathway based on physical genomic position within gene boundaries ±20 kb flanking regions to capture potential regulatory variants. Since no SNPs within autophagy- and mitophagy-related genes reached genome-wide significance (*p* < 5 × 10^−8^) in the KoGES discovery cohort, a relaxed significance threshold (*p* < 5 × 10^−5^) was applied, consistent with pathway-focused genetic studies where biological prior knowledge supports inclusion of subthreshold variants [33]. To ensure independence of genetic signals, LD pruning was performed using PLINK with an r^2^ threshold of 0.01 within 500 kb windows, yielding six independent SNPs across six autophagy-mitophagy genes.

Replication phase (UKBB): To assess whether the autophagy-mitophagy genetic architecture influences SI in an independent, ethnically distinct population, targeted genetic association analysis was conducted in the UKBB, focusing on the same CLSV-2 gene regions (19 genes ± 20 kb flanking regions) identified in KoGES. SNPs were extracted from UKBB genotype data using identical genomic coordinates to ensure consistency across cohorts. Association testing was performed using the same additive genetic model, adjusted for age, sex, assessment center, BMI, medication use, energy and alcohol intake, smoking status, and physical activity level. Assessment center was included as a geographic covariate to capture residual fine-scale population structure and regional environmental differences within the White British population.

GRS calculation: A weighted GRS was constructed by summing risk alleles weighted by their respective GWAS-derived effect sizes (β coefficients), calculated as: GRS = Σ(β_1_ × SNP_1_ + β_2_ × SNP_2_ + … + β_n_ × SNP_n_), where β_i_ represents the effect size and SNP_i_ the number of risk alleles (0, 1, or 2) for each variant. In KoGES, participants were stratified into tertiles of the weighted GRS distribution (low, medium, and high genetic risk). In UKBB, participants were classified into three categories based on GRS distribution: negative GRS (below zero), zero GRS (reference), and positive GRS (above zero).

GRS categorization: In KoGES, participants were stratified into tertiles based on the weighted GRS distribution, corresponding to low (reference), medium, and high genetic risk groups. In UKBB, the GRS distribution was approximately centered at zero due to the different SNP composition and larger sample size; therefore, participants were classified into three categories: negative GRS (below zero, lower genetic risk), zero GRS (reference), and positive GRS (above zero, higher genetic risk). While the two cohorts used overlapping but non-identical SNP sets (six in KoGES, ten in UKBB), both GRS capture genetic variation in the same autophagy-mitophagy biological pathway, enabling pathway-level replication and cross-population validation of genetic effects on SI.

### 4.9. Gene–Lifestyle Interaction Analysis

Gene–lifestyle interactions were examined using both categorical and continuous analytical approaches to assess robustness and maximize statistical power [2,9]. Categorical GRS approach: In both cohorts, participants were stratified by GRS category: tertiles (low, medium, high) in KoGES and negative/zero/positive groups in UKBB. Lifestyle factors were dichotomized based on median values or established guidelines. Logistic regression models tested interactions between categorical GRS and lifestyle factors: logit(P(SI = 1)) = β_0_ + β_1_ × GRS_category + β_2_ × Lifestyle_category + β_3_ × (GRS_category × Lifestyle_category) + covariates. Stratified prevalence estimates and interaction *p*-values were obtained. In KoGES, the Bonferroni correction was applied to account for multiple testing.

Continuous GRS approach: To maximize statistical power and capture dose–response relationships, continuous GRS and lifestyle measures were used as the primary analytical framework, particularly in UKBB, where sample size provides adequate power for interaction detection. For each lifestyle factor, logistic regression models included continuous GRS, continuous or binary lifestyle variables, and their interaction term: logit(P(SI = 1)) = β_0_ + β_1_ × GRS_continuous + β_2_ × Lifestyle + β_3_ × (GRS_continuous × Lifestyle) + covariates, where β_3_ represents the interaction effect. A negative β_3_ indicates that higher levels of the lifestyle factor attenuate genetic risk; a positive β_3_ indicates genetic risk amplification.

Covariates. All models in both cohorts were adjusted for age, sex, residential area (KoGES) or assessment center (UKBB), BMI, education level, energy intake, alcohol consumption, physical activity, smoking status, and medication use (antihypertensive, antidiabetic, lipid-lowering).

### 4.10. Statistical Analysis

All statistical analyses were performed using SAS version 9.4 (SAS Institute, Cary, NC, USA) and PLINK v2.0. Continuous variables are presented as adjusted means ± standard errors (SE), and categorical variables as frequencies and percentages. Baseline characteristics between SI cases and controls were compared using analysis of covariance (ANCOVA) for continuous variables and Chi-square tests for categorical variables, with adjustment for relevant covariates.

Multivariable logistic regression models were used to estimate associations between GRS and SI, calculating ORs and 95%CIs after adjustment for age, sex, BMI, education level, residential area, medication use, alcohol intake, smoking status, and physical activity. The reference category was the lowest GRS tertile in KoGES and the zero-GRS group in UKBB. To assess dose–response relationships, the GRS was additionally modeled as a continuous variable, with ORs calculated per unit increase in the weighted GRS.

Gene–lifestyle interactions were tested by including multiplicative interaction terms (GRS category × lifestyle factor) in logistic regression models. Lifestyle variables were dichotomized into low and high categories based on median values or clinically relevant cutoffs. A two-way ANCOVA was used to evaluate the effect of modifying lifestyle factors on the association of GRS with inflammation. To account for multiple testing across lifestyle factors, the Bonferroni correction was applied (adjusted α = 0.05/number of tests), and interactions surpassing this threshold were considered statistically significant. For significant interactions, stratified logistic regression analyses were conducted within each lifestyle stratum to estimate the GRS effects. Differences in the proportions of inflammatory cases across GRS categories were evaluated using Chi-square tests for trend.

All statistical tests were two-sided, and *p* < 0.05 was considered statistically significant unless otherwise specified. For interaction analyses, Bonferroni-corrected *p* values were used to determine statistical significance.

## 5. Conclusions

This study identifies population-level genetic associations between autophagy-mitophagy pathway variation and chronic systemic inflammation across two independent cohorts. Using pathway-based gene-set enrichment analysis, we found that the CLSV-2 autophagy-mitophagy pathway showed significant enrichment for SI associations (*p* = 0.008), with consistent cross-population replication at the gene level (*INPP5D*, *ATG7*, *VPS33A*). A weighted genetic risk score from this pathway is associated with SI and metabolic syndrome in both Asian and European ancestries. In UKBB, dietary factors—particularly coffee, fruit, and meat consumption—showed significant gene–diet interactions, with plant-based foods attenuating and meat consumption amplifying genetic risk; this is consistent with established mechanisms of dietary autophagy modulation. These observational findings complement experimental evidence linking autophagy to inflammation but have important limitations, including the use of clinical biomarkers rather than direct molecular measures, a relaxed discovery threshold in KoGES, and the inability to infer causality from cross-sectional data. Subject to validation through functional studies and intervention trials, these findings suggest that cellular quality-control pathways may represent important determinants of inflammation and that dietary strategies tailored to autophagy-mitophagy genetic profiles could inform precision approaches to inflammation prevention.

## Figures and Tables

**Table 1:** Baseline characteristics of participants according to chronic low-grade systemic inflammation (SI) status in the KoGES and UK Biobank cohorts.

	KoGES		UK Biobank	
	Low-SI (n = 18,082)	High-SI (n = 10,020)	Low-SI (n = 216,707)	High-SI (n = 127,185)
Age (years)	54.6 ± 0.07	54.1 ± 0.1 ***	56.3 ± 0.02	55.7 ± 0.03 ***
Sex (Men; N, %)	5508 (30.5)	4363 (43.5) ***	101,045 (46.6)	62,074 (48.8) ***
BMI (kg/m^2^)	23.8 ± 0.03	24.3 ± 0.04 ***	26.3 ± 0.01	27 ± 0.02 ***
Body fat (%)	27.7 ± 0.02	28 ± 0.03	29.6 ± 0.02	30.5 ± 0.02
MetS (N, %)	2106 (11.7)	2083 (20.8) ***	36,311 (16.8)	34,896 (27.4) ***
Allergy (N, %)	1279 (7.08)	688 (6.87)	66,826 (30.9)	40,311 (31.8) ***
WBC (×10^9^/L)	5.13 ± 0.009	7.36 ± 0.013 ***	5.77 ± 0.01	8.34 ± 0.01 ***
hsCRP (mg/L)	0.091 ± 0.004	0.24 ± 0.006 ***	1.07 ± 0.002	1.28 ± 0.003 ***
Alcohol (g/day)	18 ± 0.33	17 ± 0.46 ***	11.1 ± 0.05	10.1 ± 0.07 ***
Physical activity (min/day)	27.5 ± 0.22	25.9 ± 0.31 ***	58.1 ± 0.25	55.3 ± 0.33 ***
Smoking (N, %)	1246 (6.9)	1996 (20.0) ***	12,624 (5.85)	19,763 (15.6)

Values are adjusted means and standard errors, or the number and percentage of the participants. BMI, body mass index; MetS, metabolic syndrome; hsCRP, high-sensitivity C-reactive protein. Chronic low-grade systemic inflammation (SI) was defined as WBC ≥ 6.2 × 10^9^/L or hsCRP ≥ 1.0 mg/L in KoGES, and WBC ≥ 7.0 × 10^9^/L or hsCRP ≥ 3.0 mg/L in UKBB. Controls were participants below these thresholds in each cohort. Adjusted means were calculated between the groups after adjusting for covariates, including age, sex, education, residential area, BMI, energy and alcohol intake, physical activity, smoking status, and medication use. *** *p* < 0.001.

**Table 2:** Gene-set associations between Core Longevity State Vectors (CLSVs) and chronic low-grade systemic inflammation (SI) in KoGES.

	Function	N	Beta	Beta_Std	SE	*p*	Potential Function
CLSV-1	Damage tolerance	18	−0.152	−0.0048	0.1539	0.838	Prevents immune overactivation
CLSV-2	Autophagy/Mitophagy	19	0.425	0.0179	0.1766	0.008	Removes immune triggers
CLSV-3	Proteostasis	18	−0.119	−0.0037	0.1656	0.763	Prevents DAMP accumulation
CLSV-4	Basal immune readiness	18	−0.122	−0.0039	0.1977	0.732	Antiviral baseline
CLSV-5	Inflammasome restraint	17	−0.303	−0.0090	0.2074	0.928	Prevents cytokine excess
CLSV-6	Resolution capacity	19	0.053	0.0017	0.1994	0.395	Terminates inflammation

Gene list: CLSV-1: *TP53*, *BRCA1*, *BRCA2*, *ATM*, *ATR*, *RAD51*, *PALB2*, *XRCC2*, *XRCC3*, *MDC1*, *PRKDC*, *CHEK1*, *MSH2*, *MLH1*, *RPA1*, *LIG4*, *FANCA*, *FANCD2*; CLSV-2: *CDA*, *PINK1*, *DDOST*, *KIF17*, *INPP5D*, *ATG16L1*, *ATG7*, *VGLL4*, *RAB7A*, *AP3S1*, *MGAT4B*, *SQSTM1*, *MRNIP*, *TFEB*, *PRKN*, *PACRG*, *PPP2R2A*, *BNIP3L*, *OPTN*; CLSV-3: *PSMA1*, *PSMB5*, *PSMD1*, *PSMC3*, *PSME1*, *SQSTM1*, *HSP90AA1*, *HSPA1A*, *UBE2L3*, *UBB*, *UBC*, *UCHL1*, *BAG3*, *VCP*, *DNAJB1*, *HSPB1*, *PSMC6*, *PSMD2*; CLSV-4: *STAT1*, *STAT2*, *IRF3*, *IRF7*, *IFIT1*, *IFIT2*, *IFIT3*, *ISG15*, *MX1*, *OAS1*, *OAS2*, *OAS3*, *TRIM25*, *GBP1*, *IFIH1*, *IFNAR1*, *IFNAR2*, *TLR3*; CLSV-5: *NLRP3*, *NLRP1*, *PYCARD*, *MEFV*, *AIM2*, *IL1B*, *IL18*, *IL6*, *IL10*, *NLRP6*, *NLRP12*, *CASP1*, *GSDMD*, *NLRC4*, *IL1RN*, *IL37*, *IL1F10*; CLSV-6: *ALOX5*, *ALOX15*, *IL10*, *PRF1*, *GZMB*, *MERTK*, *NRP1*, *PROS1*, *ANXA1*, *IL1RN*, *CPD*, *CCR5*, *CX3CR1*, *TREM2*, *SPHK1*, *FPR2*, *ALOX12*, *PTGR1*, *LGALS9*. Beta_Std, standardized beta coefficient.

**Table 3:** Genetic variants in mitophagy and immune-autophagy (CLSV-2) genes associated with chronic low-grade systemic inflammation (SI) in KoGES and the UK Biobank.

(**A**) KoGES											
**CHR**	**SNP**	**BP**	**A1**	**A2**	**OR**	**SE**	***p***	**MAF**	**HWE**	**Gene Name**	**Location**
2	rs68147208	233978526	G	A	1.09	0.026	4.12 × 10^−5^	0.153	0.309	*INPP5D*	Intron
2	rs368068803	234169847	T	C	1.27	0.090	2.31 × 10^−5^	0.011	0.174	*ATG16L1*	Intron
3	rs149819403	11508975	T	C	0.809	0.071	3.53 × 10^−5^	0.019	0.910	*ATG7*	Intron
5	rs10055640	115244374	A	G	0.880	0.048	4.47 × 10^−5^	0.042	0.878	*AP3S1*	Intron
10	rs76421222	13153965	G	A	1.08	0.028	3.94 × 10^−5^	0.123	0.849	*OPTN*	Intron
12	rs76696405	122728704	A	G	0.870	0.047	2.88 × 10^−5^	0.043	0.190	*VPS33A*	NMD Transcript
(**B**) UK Biobank											
**CHR**	**ID**	**POS**	**A1**	**A2**	**OR**	**SE**	***p***	**MAF**	**HWE**	**Gene Name**	**Location**
2	rs7559281	234115600	T	C	0.735	0.044	2.90 × 10^−12^	0.008	0.725	*INPP5D*	3′ UTR
3	rs9848833	11446525	C	T	0.514	0.056	2.84 × 10^−32^	0.006	0.237	*ATG7*	Intron
3	rs35904610	128452108	C	T	1.079	0.013	6.75 × 10^−9^	0.086	0.865	*RAB7A*	Intron
3	rs9821206	128476739	T	C	0.786	0.043	1.48 × 10^−8^	0.008	0.154	*RAB7A*	Intron
5	rs7724740	115177262	G	C	0.732	0.037	1.49 × 10^−17^	0.011	0.513	*ATG12*	5′ UTR
5	rs10075090	179260895	A	C	0.811	0.034	1.02 × 10^−9^	0.012	0.428	*SQSTM1*	Intron
12	rs73421728	122725871	A	G	0.735	0.032	2.99 × 10^−22^	0.014	0.126	*VPS33A*	NMD transcript
15	rs2925352	41189005	G	A	0.769	0.022	2.14 × 10^−33^	0.031	0.270	*VPS18*	Intron
16	rs9972681	87425345	A	G	0.577	0.038	2.91 × 10^−47^	0.010	0.921	*MAP1LC3B*	5′ UTR
17	rs9891429	40972425	C	T	0.767	0.041	8.05 × 10^−11^	0.008	0.885	*BECN1*	Intron

CHR, chromosome; OR, adjusted odds ratio; SE, standard error; MAF, minor allele frequency. Chronic low-grade systemic inflammation (SI) was defined as WBC ≥ 6.2 × 10^9^/L or hsCRP ≥ 1.0 mg/L in KoGES, and WBC ≥ 7.0 × 10^9^/L or hsCRP ≥ 3.0 mg/L in UKBB. Controls were participants below these thresholds in each cohort. Adjusted OR was calculated between the groups after adjusting for covariates, including age, sex, education, residential area, BMI, energy and alcohol intake, physical activity, smoking status, and medication use.

**Table 4:** Interaction between GRS and lifestyles influencing chronic low-grade systemic inflammation (SI) in KoGES and the UK Biobank.

(**A**) KoGES				
	**Low-GRS** **(n = 6664)**	**Medium-GRS** **(n = 12,907)**	**High-GRS** **(n = 11,028)**	**GRS-LF Interaction** ***p* Value**
Low fat	1	1.107 (1.028–1.193)	1.273 (1.180–1.374)	0.053
High fat	1	1.305 (1.078–1.580)	1.231 (1.010–1.500)	
Low flavonoid	1	1.072 (0.986–1.165)	1.222 (1.122–1.331)	0.037
High flavonoid	1	1.289 (1.137–1.461)	1.377 (1.212–1.565)	
(**B**) UK Biobank				
	**Low-GRS** **(n = 45,176)**	**Medium-GRS** **(n = 246,990)**	**High-GRS** **(n = 51,726)**	**GRS-LF Interaction** ***p* Value**
Low BD	0.927 (0.892–0.964)	1	1.118 (1.079–1.159)	0.0053
High BD	0.931 (0.880–0.985)	1	1.019 (0.966–1.076)	
Low-Meat diet	0.898 (0.848–0.951)	1	1.059 (1.004–1.117)	0.204
High-Meat diet	0.938 (0.902–0.974)	1	1.100 (1.062–1.140)	
Low coffee	0.853 (0.798–0.911)	1	1.163 (1.091–1.241)	0.0004
High coffee	0.950 (0.915–0.985)	1	1.067 (1.032–1.103)	
Total vegetables plus fruits	0.683 (0.589–0.792)	1	1.192 (1.023–1.389)	<0.0001
	0.939 (0.909–0.971)	1	1.083 (1.050–1.116)	
Total vegetables	0.662 (0.557–0.786)	1	1.192 (1.002–1.418)	<0.0001
	0.937 (0.907–0.968)	1	1.083 (1.051–1.116)	
Total fruits	0.768 (0.685–0.861)	1	1.148 (1.031–1.279)	0.0003
	0.941 (0.910–0.973)	1	1.082 (1.049–1.116)	
Low alcohol	0.887 (0.854–0.923)	1	1.089 (1.049–1.130)	0.0034
High alcohol	0.993 (0.939–1.051)	1	1.078 (1.026–1.132)	
Low exercise	0.923 (0.872–0.977)	1	1.123 (1.064–1.185)	0.0063
High exercise	0.925 (0.890–0.962)	1	1.071 (1.034–1.110)	
Non-smoking	0.916 (0.886–0.948)	1	1.072 (1.038–1.108)	0.0013
Smoking	0.913 (0.828–1.005)	1	1.247 (1.133–1.372)	

Values are adjusted odds ratios and 95% confidence intervals. Cutoffs: Fat: 20 energy %; Flavonoids 42 mg/day; Balanced diet pattern (BD) and meat-main diet pattern (MD): 66th percentiles. Coffee intake: 0.5 cup/day; Total vegetables + fruits: 2 servings/day; Total veg: 1 serving/day; Total fruit: 1 serving/day; alcohol: 7.9 g/day; exercise: 30 min/day. Chronic low-grade systemic inflammation (SI) was defined as WBC ≥ 6.2 × 10^9^/L or hsCRP ≥ 1.0 mg/L in KoGES, and WBC ≥ 7.0 × 10^9^/L or hsCRP ≥ 3.0 mg/L in UKBB. Controls were the participants below these thresholds in each cohort. Adjusted OR was calculated between the groups after adjusting for covariates, including age, sex, education, residential area, BMI, energy and alcohol intake, physical activity, smoking status, and taking medication. LF, lifestyles.

## Data Availability

The original contributions presented in this study are included in the article and Supplementary Materials. Further inquiries can be directed to the corresponding authors.

## Supplementary Materials

The following supporting information can be downloaded at: https://www.mdpi.com/article/10.3390/ijms27073062/s1.
