# Supplementary material for: Autophagy-Mitophagy Pathway-Linked Genetic Variants Associate with Systemic Inflammation and Interact with Dietary Factors in Asian and European Cohorts
Source: Int J Mol Sci. 2026 Mar 27;27(7):3062. doi: 10.3390/ijms27073062 (PMC13072760; doi:10.3390/ijms27073062)
Supplement: Supplementary file 1 [file ijms-27-03062-s001.zip › ijms-4207899-supplementary.pdf]

Supplementary Figures

Figure S1. Flow chart of participant inclusion and experimental procedure for determining genetic variants for chronic low-grade systemic inflammation (SI) risk

A. Korean Genome and Epidemiology Study (KoGES)
B. UK Biobank

Figure S2. Genetic variant distribution for chronic low-grade systemic inflammation (SI) risk in a genome-wide association study in the Korean Genome and Epidemiology Study (KoGES).

A. Manhattan plot of the p-values of genetic variants. The red dotted line indicates the p-value of the cutoff of genetic variants for SI risk.
B. Q–Q plot of observed and expected p-values for KoGES. The red dotted line indicates the calculated observed and expected p-values. It indicates the matching between observed and expected p-values.

(A)

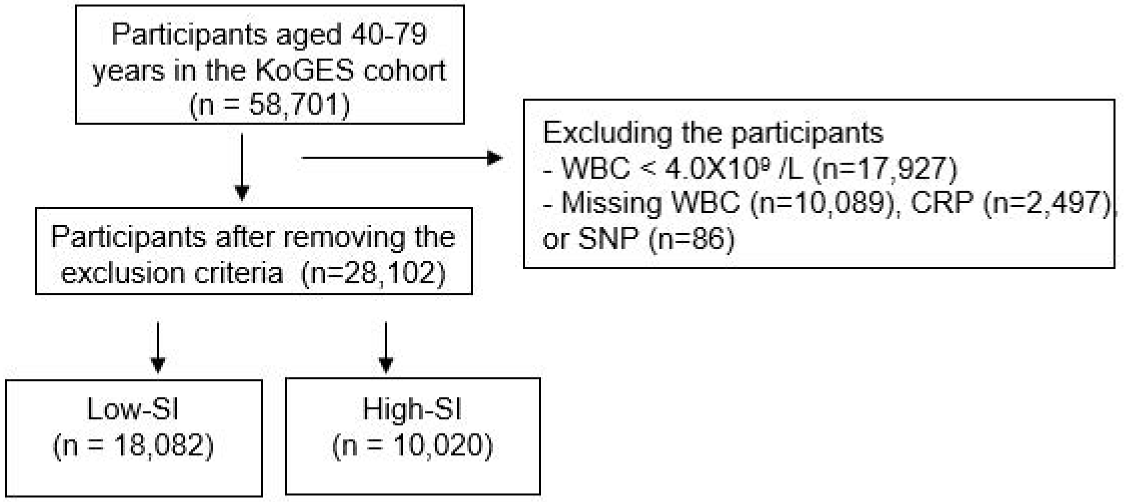
Participants aged 40-79 years in the KoGES cohort (n = 58,701)
Excluding the participants
- WBC < 4.0X10⁹ /L (n=17,927)
- Missing WBC (n=10,089), CRP (n=2,497), or SNP (n=86)
Participants after removing the exclusion criteria (n=28,102)
Low-SI (n = 18,082)
High-SI (n = 10,020)

(B)

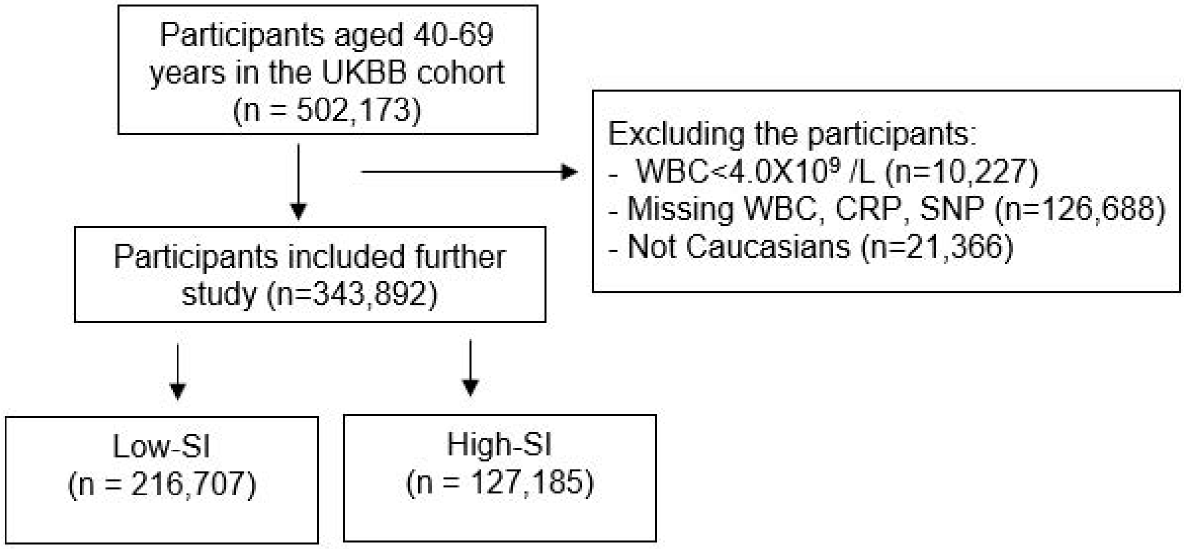
Participants aged 40-69 years in the UKBB cohort (n = 502,173)
Excluding the participants:
- WBC<4.0X10⁹ /L (n=10,227)
- Missing WBC, CRP, SNP (n=126,688)
- Not Caucasians (n=21,366)
Participants included further study (n=343,892)
Low-SI (n = 216,707)
High-SI (n = 127,185)

Figure S1.

(A)

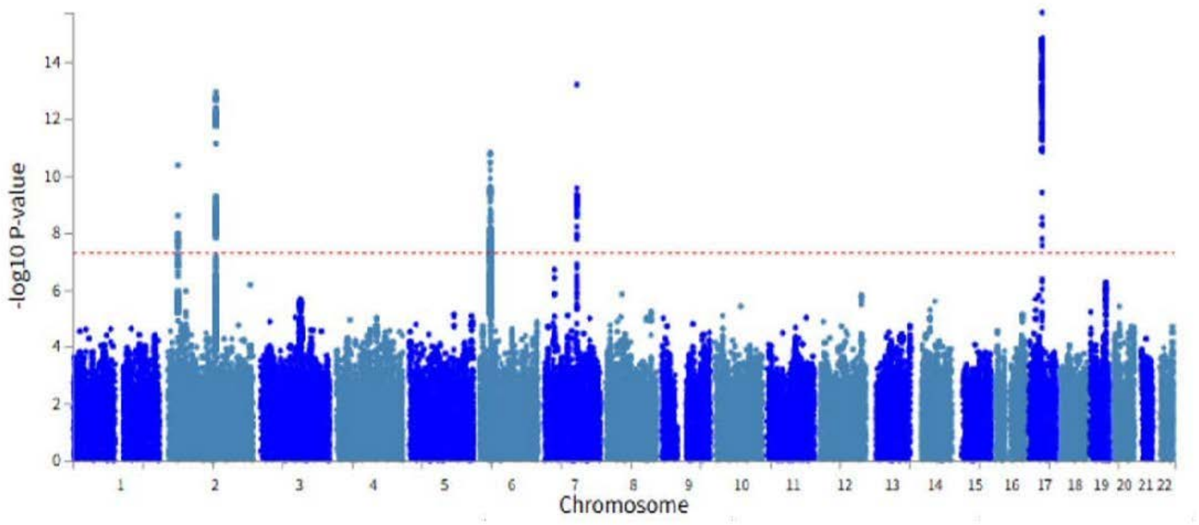

-log10 P-value
Chromosome

(B)

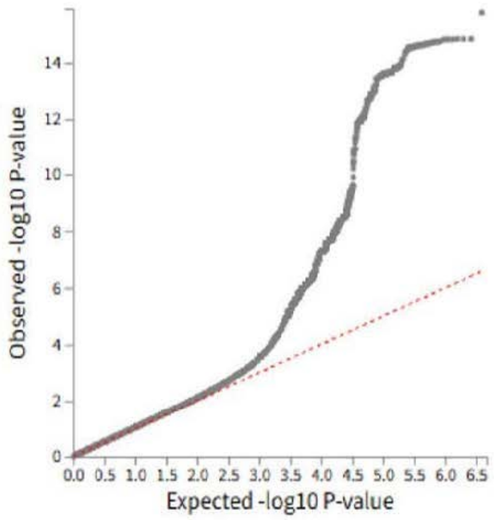

Observed -log10 P-value
Expected -log10 P-value

Figure S2.

Supplementary Tables

Table S1. Gene-Lifestyle Interaction Analysis in UK Biobank

Factor	Variable Type (Units)	Main Effect: GRS β (95% CI)	Main Effect: Lifestyle β (95% CI)	Interaction β (95% CI)	P-interaction
**DIETARY FACTORS**					
Coffee consumption	Continuous (cups/day)	0.130 (0.114-0.146)	0.015 (0.010-0.020)	-0.010 (-0.018 to -0.003)	0.006
Total fruit intake	Continuous (servings/day)	0.127 (0.109-0.146)	-0.034 (-0.038 to -0.029)	-0.004 (-0.008 to -0.0002)	0.039
Total vegetable intake	Continuous (servings/day)	0.122 (0.103-0.140)	-0.009 (-0.012 to -0.006)	-0.002 (-0.004 to 0.001)	0.224
Vegetable + fruit combined	Continuous (servings/day)	0.129 (0.107-0.150)	-0.014 (-0.017 to -0.012)	-0.002 (-0.004 to -0.0001)	0.040
Meat intake	Continuous (servings/day)	0.107 (0.093-0.120)	0.086 (0.074-0.098)	+0.023 (0.013-0.034)	<0.001
**OTHER LIFESTYLE FACTORS**					
Alcohol consumption	Continuous (drinks/day)	0.121 (0.108-0.135)	-0.004 (-0.005 to -0.003)	-0.001 (-0.002 to 0.0004)	0.305
Physical activity	Continuous (total activity/day)†	0.112 (0.097-0.127)	-0.00007 (-0.00009 to -0.00005)	0.00002 (-0.00001 to 0.00005)	

Continuous gene-lifestyle interaction analyses for systemic inflammation in UK Biobank data (n=343,892). Models: logit(SI) = $\beta_0 + \beta_1 \times GRS + \beta_2 \times Lifestyle + \beta_3 \times (GRS \times Lifestyle)$ + covariates. Main Effect GRS (β_1): effect of genetic risk score when lifestyle variable = 0. Main Effect Lifestyle (β_2): effect of lifestyle independent of genetics. Interaction (β_3): change in GRS effect per unit increase in lifestyle variable. Negative β_3 indicates lifestyle attenuates genetic risk; positive β_3 indicates lifestyle amplifies genetic risk. Covariates: age, sex, assessment center, BMI, energy intake, medication use, and relevant lifestyle factors (dietary models adjusted for alcohol, physical activity, smoking; alcohol/physical activity models adjusted for diet). GRS = weighted genetic risk score from autophagy-mitophagy pathway SNPs. SI = white blood cell count $>7.0\times10^9$/L or C-reactive protein >3.0 mg/L. Bold indicates P<0.05. †MET-minutes/day

Table S2A. GRS associations with chronic low-grade systemic inflammation (SI) under alternative definitions in KoGES

SI Definition	SI prevalence	Middle GRS vs Low	High GRS vs Low
	N (%)	OR (95% CI)	OR (95% CI)
**Primary analysis**			
WBC >6.2 *or* CRP >1.0	10,020 (35.7%)	1.27 (1.18-1.36)	1.13 (1.06-1.22)
**Sensitivity: Single-marker definitions**			
WBC >6.2 only	9,914 (35.3%)	1.09 (1.02-1.16)	1.19 (1.12-1.27)
CRP >1.0 only	483 (1.72%)	1.01 (0.79-1.30)	1.06 (0.82-1.36)
**Sensitivity: Quartile-based definition**			
WBC >6.51 *or* CRP >0.118	12,000 (42.7%)	1.05 (0.99-1.11)	1.10 (1.03-1.17)

Table S2B. Continuous inflammatory marker outcomes in KoGES and UKBB

**KoGES**				
Outcome	Low GRS (Group 1)	High GRS (Group 2)	Difference (95% CI)	P-value
	LSMean (95% CI)	LSMean (95% CI)		
WBC ($\times 10^9$/L)	5.13 (5.12-5.15)	7.36 (7.34-7.39)	+2.23 (2.20-2.26)	<0.001
CRP (mg/L)	0.091 (0.082-0.100)	0.237 (0.224-0.249)	+0.146 (0.131-0.161)	<0.001
log(CRP)	-2.74 (-2.75 to -2.72)	-2.30 (-2.33 to -2.28)	+0.43 (0.40-0.46)*	<0.001
UKBB				
WBC ($\times 10^9$/L)	5.77 (5.76-5.78)	8.34 (8.33-8.35)	+2.57 (2.56-2.58)	<0.001
CRP (mg/L)	1.07 (1.07-1.08)	1.28 (1.27-1.29)	+0.21 (0.20-0.22)	<0.001
log(CRP)	0.012 (0.008-0.017)	0.032 (0.026-0.038)	+0.18 (1.20-fold)†	<0.001

*Corresponds to 1.54-fold higher CRP in high GRS group (95% CI: 1.49-1.59)

Table S2C. Chronic low-grade systemic inflammation (SI)-metabolic syndrome (MetS) associations under alternative definitions in KoGES

SI Definition	SI prevalence (N, %)	MetS among SI (N, %)	OR (95% CI)	P-value
**Primary analysis**				
WBC >6.2 *or* CRP >1.0	10,020 (35.7%)	2,083 (20.8%)	1.65 (1.52-1.78)	<0.001
**Sensitivity: Single-marker definitions**				
WBC >6.2 only	9,914 (35.3%)	2,664 (26.9%)	1.65 (1.54-1.77)	<0.001
CRP >1.0 only	483 (1.72%)	102 (21.1%)	1.14 (0.92-1.40)	0.23
**Sensitivity: Independent quartile-based**				
WBC >6.51 *or* CRP >0.118†	12,000 (42.7%)	3,049 (21.4%)	1.63 (1.52-1.74)	<0.001

†Quartile-based definition derived independently of metabolic syndrome. GRS = Genetic risk score from autophagy-mitophagy pathway SNPs (categorized as low/middle/high risk groups) All GRS models and SI→MetS models adjusted for age, sex, education level, residential area, BMI, medication use (antihypertensive, antidiabetic, and lipid-lowering agents), energy and alcohol intake, smoking status, and physical activity level. The quartile-based SI definition uses the 75th percentile cutoffs (WBC >6.51×10^9/L or CRP >0.118 mg/L), derived independently of metabolic syndrome to assess potential circularity.
